# Hypervirulence-associated pseudo-compound transposons as fundamental mobile units driving cross-species virulence dissemination in Enterobacteriaceae

**DOI:** 10.1371/journal.ppat.1014513

**Published:** 2026-09-01

**Authors:** Shuaihua Fan, Lijun Wang, Chao Liu, Haoran Li, Huaiqing Qi, Pengcheng Du, Jun Guo

**Affiliations:** 1 Department of Geriatric, Beijing Tsinghua Changgung Hospital, School of Clinical Medicine, Tsinghua Medicine, Tsinghua University, Beijing, China; 2 Department of Laboratory Medicine, Beijing Xiaotangshan Hospital, Beijing, China; 3 Department of Infectious Disease, Peking University Third Hospital, Beijing, China; 4 Medical Research Center, Beijing Institute of Respiratory Medicine and Beijing Chao-Yang Hospital, Capital Medical University, Beijing, China; INSERM U1220, FRANCE

## Abstract

**Background:**

The rapid global spread of hypervirulence in Enterobacteriaceae, particularly in carbapenem-resistant *Klebsiella pneumoniae*, poses a significant public health threat. However, the key genetic vehicles and mechanisms driving horizontal transfer of hypervirulence-associated genes (*iucA*, *iroB*, *rmpA*, *rmpA2*, and *peg-344*) remain poorly defined, limiting effective surveillance.

**Methods:**

We performed a large-scale genomic survey of 2,869 virulence-associated plasmid sequences and 2,337 complete Enterobacteriaceae chromosomes. Using comparative genomics and evolutionary analyses, we systematically identified and characterized Hypervirulence-associated Pseudo-Compound Transposons (Hva-PCTs), defined as structured mobile elements in which hypervirulence-associated genes are flanked by insertion sequences.

**Results:**

Our results demonstrate that hypervirulence-associated genes are transmitted primarily as discrete IS-bounded units, which we term Hva-PCTs. We identified 29 distinct plasmid-borne Hva-PCTs (pHva-PCTs) and 30 chromosomal Hva-PCTs (cHva-PCTs). These modules show clear species-specific patterns: *iucA/iroB*-associated Hva-PCTs mainly originate in *Escherichia coli* and spread through IncFIB-containing multi-replicon plasmids (commonly combined with IncFIC(FII) and/or IncFII, while *rmpA/rmpA2/peg-344*-containing modules originate in *K. pneumoniae* and are disseminated via IncHI1B/repB plasmids. Three Hva-PCTs were detected on both plasmids and chromosomes (xHva-PCTs). In one clinical *K. pneumoniae* isolate (LS356), the identical composite module was present on both replicons. Simpler sub-modules, such as IS*Kqu3-rmpA2-iucA_1*-IS*102* and IS*102-rmpA-peg-344-iroB_1*-IS*1A*, frequently co-occur on the same plasmid; when positioned in tandem, they reconstitute the full composite structure. This assembly pattern is further supported by a partial duplication event in plasmid pP901. CD-HIT clustering (80% nucleotide identity and 90% coverage) showed that 13 of 22 major clusters contained both plasmid and chromosomal copies, with intra-cluster identities >80% across multiple sequence types and host species.

**Conclusion:**

Hypervirulence-associated genes in Enterobacteriaceae are disseminated mainly as IS-flanked Hva-PCTs rather than solely through intact virulence plasmids. These modules exhibit strong but not absolute host specificity. The presence of identical Hva-PCTs on plasmids and chromosomes suggests inter-replicon mobility, while their stepwise assembly from simpler sub-modules highlights modular accretion as a key evolutionary process. Tracking Hva-PCTs as distinct mobile units may complement existing plasmid- and gene-centric surveillance approaches for hypervirulent and convergent strains. Experimental validation of their transposition activity and phenotypic effects is still required.

## Author summary

Bacteria swap genes like tools, and some of those genes make them more dangerous. Hypervirulent *Klebsiella pneumoniae* can cause severe infections even in healthy people, and the hypervirulence-associated genes responsible are spreading fast—but how they move has been unclear. We scanned nearly 20,000 Enterobacteriaceae genomes and tracked five hypervirulence-associated genes across plasmids, chromosomes, and species. Rather than traveling as part of intact virulence-associated plasmids, these genes move in compact cassettes flanked by insertion sequences—structures we named Hva-PCTs. Different species carry different types: *E. coli* prefers simple iron-scavenging modules on IncFIB-based multi-replicon plasmids, while *K. pneumoniae* carries more complex modules combining iron genes with capsule regulators on IncHI1B/repB-based multi-replicon plasmids. Some cassettes appear in identical form on both plasmids and chromosomes, sometimes in the same strain, suggesting they can jump between replicon types. Simpler cassettes also recombine into more complex ones. Because these cassettes, not entire plasmids, are the real moving parts, tracking them could sharpen surveillance of emerging hypervirulent and drug-resistant strains. Experimental tests are still needed to confirm their mobility and effects.

## Introduction

The opportunistic pathogen *Klebsiella pneumoniae* can cause serious infections and lead to life-threatening diseases, imposing an increasing infection burden worldwide [1–3]. Based on phenotypic and genotypic characteristics, *K. pneumoniae* can be categorized into two types: classic *K. pneumoniae* (cKP) and hypervirulent *K. pneumoniae* (hvKP) [4, 5]. While cKP strains are typically associated with healthcare settings and multidrug resistance [6], hvKP exhibits markedly enhanced virulence and frequently causes invasive community-acquired infections even in otherwise healthy individuals [5]. The hypervirulent phenotype is closely linked to several key virulence factors, most notably the aerobactin siderophore locus (*iucABCD-iutA*), the salmochelin locus (*iroBCDN*), the mucoid phenotype regulators *rmpA* and *rmpA2*, and the marker gene *peg-344* [4, 7]. These loci are widely used as molecular indicators for identifying hvKP and assessing virulence potential in clinical isolates [4].

Although the epidemiology and clinical impact of hvKP have been well documented, the genetic mechanisms governing the dissemination and stable maintenance of hypervirulence remain only partially understood. In contrast to antimicrobial resistance genes [8, 9], the mobilization pathways of hypervirulence-associated loci have received far less systematic attention. Earlier studies have largely focused on a plasmid-centric view, noting that many hvKP isolates carry large virulence plasmids harboring *iuc, iro, rmpA/rmpA2*, and related genes [10–14]. However, accumulating genomic evidence indicates that whole plasmids may not always be the most informative unit for tracking hypervirulence spread. Instead, virulence loci are frequently embedded in smaller, IS-shaped mobile regions that undergo local rearrangements, recombination, and transposition [13, 15, 16].

Insertion sequences (ISs) are among the most abundant mobile genetic elements in bacteria and play critical roles in transposition, recombination, deletion, inversion, duplication, and the formation of composite or pseudo-compound transposon-like structures [17, 18]. In antimicrobial resistance research, IS-flanked modules are recognized as major drivers of horizontal gene transfer across plasmids, chromosomes, and species boundaries [9, 19, 20]. By comparison, whether hypervirulence-associated genes in Enterobacteriaceae follow similar modular mobilization patterns has not been comprehensively examined. This is particularly relevant because virulence gene dissemination may extend beyond plasmid-to-plasmid transfer: IS-associated regions can also move between plasmids and chromosomes, integrate into new genomic contexts, and undergo lineage-specific adaptation [18, 21, 22].

Recent comparative analyses have shown that hypervirulence-associated loci are not restricted to *K. pneumoniae*; they also appear in *Escherichia coli, Klebsiella variicola, Enterobacter spp.*, and *Citrobacter spp.*, pointing to a broader interspecies gene pool [7, 23–26]. Virulence plasmids themselves are structurally dynamic, frequently experiencing insertions, deletions, rearrangements, and recombination during evolution and transmission [14, 27, 28]. These findings suggest that cross-species spread of hypervirulence depends not only on the transfer of entire plasmids but also on the successful mobilization, maintenance, and functional integration of individual virulence loci or linked modules into new plasmid or chromosomal backgrounds.

Among these loci, the *iuc* and *iro* clusters are especially suitable for investigating modular dissemination [7]. They encode high-affinity siderophore systems that confer a fitness advantage under iron-limited conditions in the host and have demonstrated functional importance beyond *K. pneumoniae*, including contributions to extraintestinal virulence in *E. coli* and other Enterobacteriaceae [7, 29, 30]. In contrast, the contribution of *rmpA/rmpA*2 to virulence appears more dependent on the recipient strain’s genomic background, particularly capsule biosynthesis and regulatory compatibility [5, 31, 32]. These differences make the *iuc/iro* loci useful models for studying modular transfer, while *rmpA/rmpA2* offers a valuable contrast for understanding host-specific constraints.

To address these gaps, we conducted a large-scale comparative genomic analysis of Enterobacteriaceae focusing on five canonical hypervirulence-associated genes: *iucA, iroB, rmpA, rmpA2*, and *peg-344*. Moving beyond the traditional plasmid-centric framework, we examined these genes together with their flanking IS elements and local genetic contexts. Our objectives were to (1) define the core IS-associated mobile units responsible for hypervirulence gene transmission, (2) characterize their structural diversity, genomic distribution, and species specificity, and (3) reconstruct the routes of transmission and integration across plasmids and chromosomes. This module-level approach offers a genome-wide perspective on how hypervirulence loci spread through both plasmid-mediated transfer and plasmid-chromosome exchange within Enterobacteriaceae.

## Methods

### Bacterial genome sequences

We retrieved 19,601 complete Enterobacteriaceae genome assemblies from NCBI GenBank (accessed May 2025). Only assemblies annotated as “Complete Genome” or “Chromosome” were retained to reduce fragmentation artifacts. Missing isolate metadata (isolation time, source, and geographic location) were manually curated from associated publications. Multilocus sequence typing (MLST) was performed using MLST v2.0 (https://github.com/tseemann/mlst) with default settings. Species distribution and genome counts are summarized in S1 Table.

### Identification of Hypervirulence-associated Isolates

Virulence-associated plasmids were defined as those carrying at least one of the five key hypervirulence-associated genes of *K. pneumoniae*: *iucA, iroB, rmpA, rmpA2,* and *peg-344*. The *iucA* and *iroB* genes were detected using Kleborate database via BLAST 2.15.0+ [33] at 90% nucleotide identity and 80% coverage thresholds. The *rmpA* and *rmpA2* genes were identified by BLAST searches against the Virulence Factor Database [34] using the same thresholds. The *peg-344* gene was detected by direct BLASTn querying with its reference sequence. HvKP was defined as those harbored at least one of the five hypervirulence-associated genes of *K.pneumoniae*.

### Genome annotation

Chromosomal and plasmid sequences were separated using SeqKit [35]. Both datasets were annotated with Prokka [36]. Insertion sequences (ISs) were identified using ISfinder [37], and plasmid replicon types were determined using PlasmidFinder [38]. Plasmids carrying two or more distinct replicon types were classified as multi-replicon plasmids.

### Phylogenetic analysis and comparative genomic analysis

For each hypervirulence-associated gene, coding sequences were extracted using SeqKit. Multiple sequence alignment was performed with MAFFT [39]. The maximum likelihood phylogeny was inferred using IQ-TREE (version 2.4.0) [40]. The best-fit substitution model was selected using ModelFinder [41], and branch support was evaluated with 1000 ultrafast bootstrap replicates [42]. The phylogenetic trees were visualized and annotated using iTOL (https://itol.embl.de/). Phylogenetic clades were delineated based on genetic distance and bootstrap (>70%). From each major branch, three to five representative isolates were selected for downstream analysis.

### Identification and classification of Hypervirulence-associated Pseudo-Compound Transposons (Hva-PCTs)

Hva-PCTs were defined as discrete genetic modules containing at least one hypervirulence-associated gene and bounded by insertion sequences. An IS was considered flanking if located within 12 kb upstream or downstream of the target gene (a distance chosen to capture typical composite transposon structures while excluding distant elements). Hva-PCTs were categorized as plasmid-borne (pHva-PCTs), chromosomal (cHva-PCTs), or cross-replicon (xHva-PCTs) according to their genomic location. Conservation between plasmid and chromosomal copies was evaluated by pairwise BLASTn, reporting nucleotide identity and query coverage. Flanking genetic contexts were visualized using Easyfig [43]. To investigate evidence of Hva-PCT dissemination, we performed clustering analysis on all extracted Hva-PCT sequences using the cd-hit-est (version 4.8.1) module with thresholds of 80% nucleotide identity and 90% alignment coverage.

### Identification of co-evolutionary relationships among virulence genes

We conducted Mantel tests to assess the degree of correlation between phylogenetic distance matrices derived from each gene's evolutionary history using the vegan package (version 2.7-1) in R. A patristic distance matrix was generated for each gene from its phylogenetic tree using the cophenetic.phylo() function in the ape package (version 5.8). This matrix quantifies the evolutionary divergence (sum of branch lengths) between all pairs of bacterial strains for that specific gene. Pairwise Mantel tests with 9,999 permutations were then used to calculate the Pearson correlation coefficient between each pair of gene patristic distance matrices. This test determines whether the evolutionary history of one gene is significantly correlated with that of another, which would be indicative of co-evolution. The resulting correlation coefficients and their statistical significance were compiled into a matrix and visualized as a heatmap using the corrplot package (version 0.92).

We distinguished two types of gene associations: “co-location” refers to the physical linkage of two or more genes on the same contiguous DNA segment (e.g., within a transposon or genomic island), whereas “co-occurrence” denotes the presence of multiple genes within the same genome regardless of their genomic positions. This distinction was maintained throughout subsequent analyses to differentiate modular mobility from overall strain virulence potential.

### Statistical analysis and visualization

Statistical analysis was conducted using R version 4.1.0. Essential packages facilitate data handling, visualization, and sequence analysis. Ggplot2 (version 3.5.1) was used to generate pie charts, genomic composition plots, and Sankey plot. Data manipulation was streamlined using dplyr (version 1.1.4), while the tidyverse (version 2.0.0) ensured a cohesive workflow throughout the analysis. Complex datasets were visualized using Pheatmap (version 1.0.12) for heat maps.

## Results

### Hypervirulence-associated genes form structured, species-specific groups in Enterobacteriaceae

**Fig 1:**
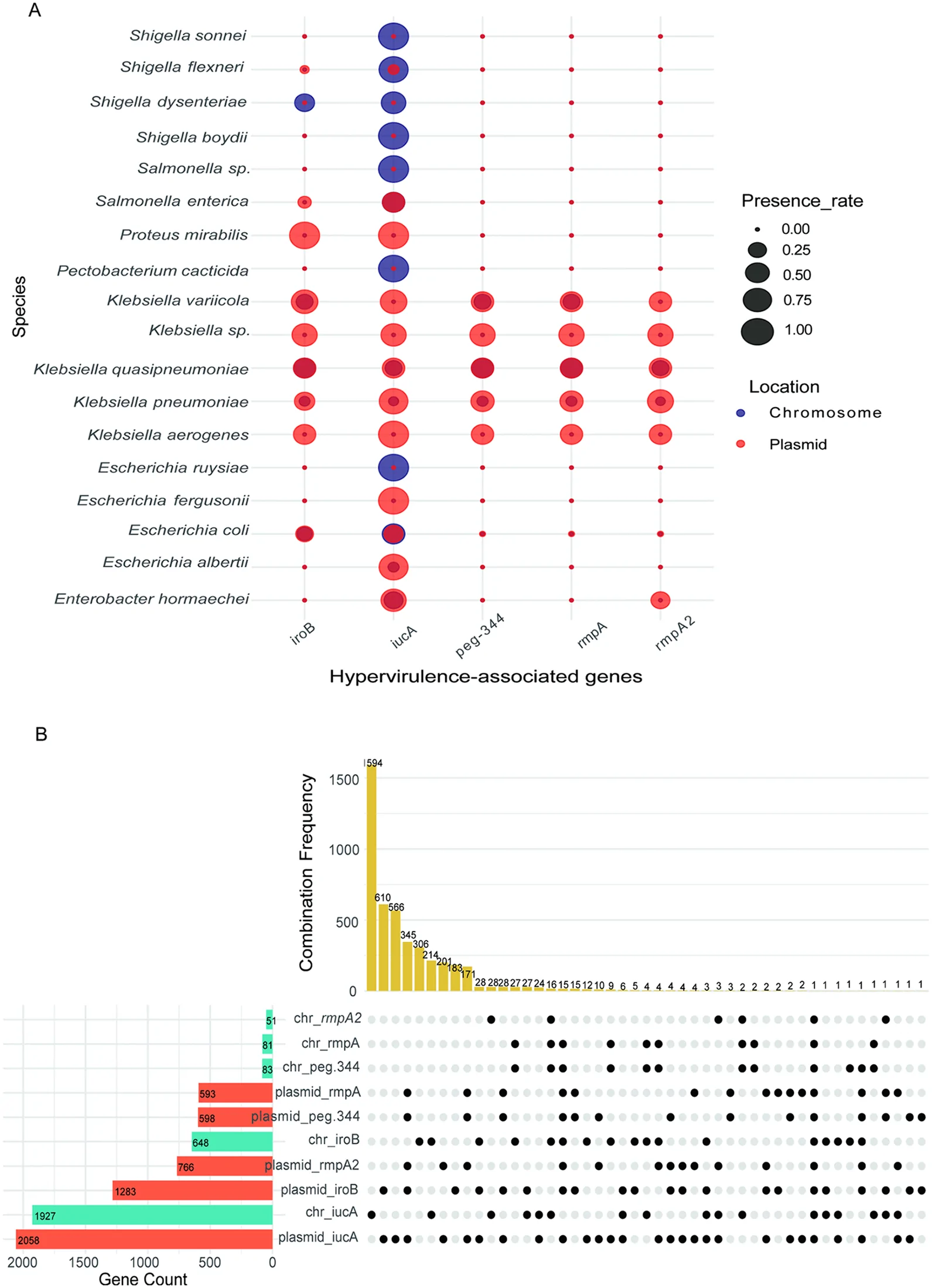
Species-specific distribution and genetic localization of hypervirulence-associated genes across Enterobacteriaceae. **(A)** Bubble plot illustrating the prevalence and genomic location of the five hypervirulence-associated genes across major Enterobacteriaceae species. Each bubble size represents the percentage of isolates carrying within a given specific harbouring the corresponding gene. The colour of each bubble indicates the predominant genetic location of the gene (chromosome or plasmid) within that species. **(B)** Stacked bar chart on the left showing the frequency of the top 10 most common genetic contexts in which the hypervirulence-associated genes were identified. Each bar represents a specific gene-location combination, e.g., *rmpA*2 (C) denotes the *rmpA*2 gene located on the chromosome and *rmpA2* (P) means on the plasmid, with the total number of isolates for each combination labelled above the bars. The histogram on the top represents the frequency of each hypervirulence-associated gene combination.

After quality control and manual investigation of available genome assemblies from NCBI GenBank, we established a comprehensive dataset of 19,601 Enterobacteriaceae genomes, including 7,084 *E. coli* (36.1%), 4,392 *K. pneumoniae* (22.4%), 3,134 *Salmonella enterica* (16.0%), 158 *Shigella flexneri* (0.8%), and 36 *Shigella dysenteriae* (0.18%) genomes. Among these genomes, 4,491 (22.9%) carried at least one hypervirulence-associated gene (*iroB, iucA, rmpA, rmpA2,* and *peg-344*) (S2 Table), 67.3% (3,021/4,491) from *E. coli*, 23.1% (1,039/4,491) from *K. pneumoniae*, 2.8% (126/4,491) from *Shigella flexneri*, 2.6% (115/4,491) from *Salmonella enterica*, and 0.7% (32/4,491) from *Shigella dysenteriae*. The prevalence of the five genes varied substantially across the dataset: *iroB* was detected in 1,877/4,491 isolates (41.8%), *iucA* in 3,946/4,491 (87.9%), *rmpA* in 658/4,491 (14.7%), *rmpA2* in 813/4,491 (18.1%), and *peg-344* in 664/4,491 (14.8%) (Fig 1A, 1B).

Further analysis shows that the distribution of these genes exhibited significant species-specific and locus-specific patterns (Figs 1B, S1A, S1B, and S3 Table). *IroB* and *iucA* exhibited a broader taxonomic distribution, while the genes *rmpA, rmpA2*, and *peg-344* were predominantly confined to the genus *Klebsiella*, with more than 90% specifically identified in *K. pneumoniae* (S2A Fig). Among the 1,039 hvKP isolates, all five hypervirulence-associated genes were prevalent and exhibited a strong preference for plasmid carriage: *iroB* (45.2% prevalence; 87.7% plasmid-borne), *iucA* (94.8%; 94.6%), *rmpA* (60.4%; 90.1%), *rmpA2* (75.4%; 94.1%), and *peg-344* (61.1%; 90.1%). In contrast, *E. coli* exhibited different localization patterns for the five genes: *iroB* was primarily plasmid-borne (61.8%), while *iucA* was predominantly chromosomal (61.3%). The *rmpA, rmpA2,* and *peg-344* genes were rarely detected in *E. coli* (≤0.7%), and when present, they were almost exclusively plasmid-borne.

### Co-evolution and co-localization of hypervirulence-associated genes define Hva-PCTs

*RmpA, rmpA2*, and *peg-344* are largely restricted to the *Klebsiella*. To understand dissemination across the broader Enterobacteriaceae, we first focused on the widely distributed *iroB* and *iucA* loci. Although *K. pneumoniae* had fewer STs (n = 112) than *E. coli* (n = 280) in the dataset, it showed greater diversities of *iroB* and *iucA* across both chromosomes and plasmids (S2B and S2C Fig). In *E. coli*, four *iroB* variants were identified: *iroB*_*23* (61.6%), *iroB*_*31* (35.1%), *iroB*_*27* (2.1%) and *iroB*_*1* (1.3%). Similarly, four *iucA* variants were identified (*iucA_1*, *iucA_29, iucA_38, iucA_45*), predominantly *iucA*_*38* (62.5%) and *iucA*_*45* (34.0%). In contrast, *K. pneumoniae* carried 13 *iroB* variants (*iroB_1* at 78.6% and *iroB_6* at 8.9% being the most prevalent) and 23 *iucA* variants (*iucA_1* at 78.3%). To trace potential transmission pathways of these variants, we analyzed their distribution across genetic loci (chromosome vs. plasmid) and host species. Host-specific patterns emerged: *E. coli* plasmids predominantly carried the *iroB_23-iucA_45* combination (44.4%), while chromosomes primarily hosted *iroB_31-iucA_38* (11.2%). Rare chromosomal co-occurrence of *iucA_45* with *iroB_23* (0.3%) or *iroB_27* (0.05%) was observed. In contrast, *K. pneumoniae* strongly preferred the *iucA_1-iroB_1* combination, which was predominantly plasmid-encoded (34.8%) with limited chromosomal presence (12.2%).

**Fig 2:**
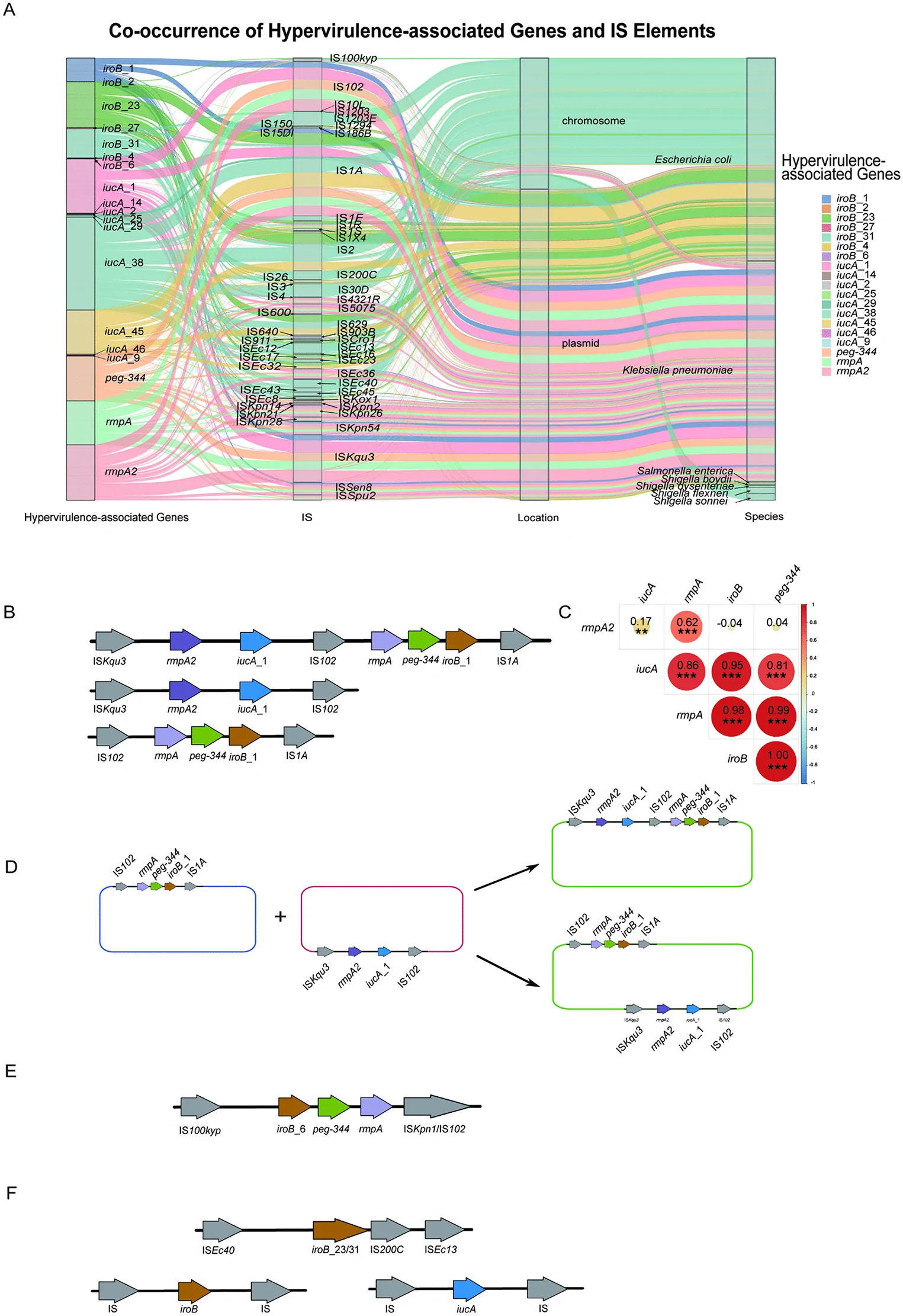
Genomic architecture and proposed reassembly mechanism of Hypervirulence-associated pseudo-compound transposons (Hva-PCTs). **(A)** Alluvial plot illustrating the co-occurrence network among hypervirulence-associated genes, insertion sequence (IS) elements (middle), genetic location (chromosome or plasmid), and species. **(B and D)** Schematic depiction of the three cross-replicon Hva-PCTs (xHva-PCTs) and their recombination relationships. **(B)** Structural organization of the three xHva-PCT types (type 1: full composite module; types 2 and 3: simpler sub-modules). The frequent co-occurrence of types 2 and 3 on the same plasmid supports a stepwise assembly model in which recombination between these sub-modules reconstitutes the full type 1 structure, as illustrated in **(D)**. These modules are primarily plasmid-borne in *K. pneumoniae*, with additional chromosomal copies also observed. **(C)** Co-evolutionary matrix analysis of hypervirulence-associated genes. The matrix illustrates significant pairwise evolutionary correlations among the five hypervirulence-associated genes as determined by Mantel tests. Node size corresponds to the strength of correlation (absolute r value), while node colour indicates positive (red) or negative (blue) correlation. **(E)** A cHva-PCT carrying multiple hypervirulence-associated genes, which is mainly located on the chromosome of *K. pneumoniae*, though it has also been detected in *E. coli*. **(F)** In addition to the above multi-virulence gene-carrying Hva-PCTs, *iroB* and *iucA* can form Hva-PCTs with various IS elements, facilitating the spread of hypervirulence-associated genes. These modules are primarily found in *E. coli*.

To directly test for historical cross-species transmission events, we performed phylogenetic analysis on all five core hvKP-associated genes (including *rmpA*, *rmpA2*, and *peg-344*). The trees, constructed from 2,337 chromosomal and 2,869 plasmid-borne sequences, confirmed interspecies transmission, with shared variants forming mixed-species clusters (S3 and S4 Figs). This evidence of cross-host dissemination, combined with the species-specific co-occurrence patterns of the five genes, suggests that these genes do not evolve independently. Instead, they likely co-evolve and spread as integrated units (S3 Table). To quantify the evolutionary linkage among these genes, we performed Mantel tests (Fig 2C). The results showed high correlation indices for *iroB-peg-344* (1.00, P < 0.001), *iroB-iucA* (0.95, P < 0.001), *iroB-rmpA* (0.98, P < 0.001), *rmpA-peg-344* (0.99, P < 0.001), *iucA-peg-344* (0.81, P < 0.001), and *rmpA-rmpA2* (0.62, P < 0.001). *RmpA2* also showed a weaker but significant correlation with *iucA* (P < 0.01). These findings are consistent with the co-occurrence patterns of the five genes (S3 Table). The observed co-localization and co-evolutionary patterns indicate that these hypervirulence-associated genes disseminate as coordinated genomic modules rather than independently.

Furthermore, the five hypervirulence-associated genes showed a significant tendency for co-localization with specific flanking IS elements (Fig 2A). Phylogenetic and co-evolutionary analyses further revealed that these genes and their cognate IS elements form evolutionarily stable genetic modules. Based on these structural and evolutionary properties, we define these discrete IS-flanked modules as Hypervirulence-associated Pseudo-Compound Transposons (Hva-PCTs) (Fig 2B, 2D-2F).

### Hva-PCTs display species-specific and plasmid type-specific distrubutions

From 2,337 chromosomes and 2,869 plasmids, we identified 29 distinct plasmid-borne Hva-PCTs (pHva-PCTs) and 30 chromosomal Hva-PCTs (cHva-PCTs) (S4 Table). Most plasmids (1,801/2,706, 66.6%) and chromosomes (2,001/2,477, 80.8%) carried only a single Hva-PCT (S5 Table); genomes without any Hva-PCT were excluded from the denominator. Among the Hva-PCT-carrying plasmids, 20 lacked a detectable replicon type. Of the remainder, 5.6% (151/2,706) were single-replicon and 93.6% (2,535/2,706) were multi-replicon. A few variants showed strict plasmid-type associations, though all occurred at low frequency (maximum count: 23) (S4 Table).

**Fig 3:**
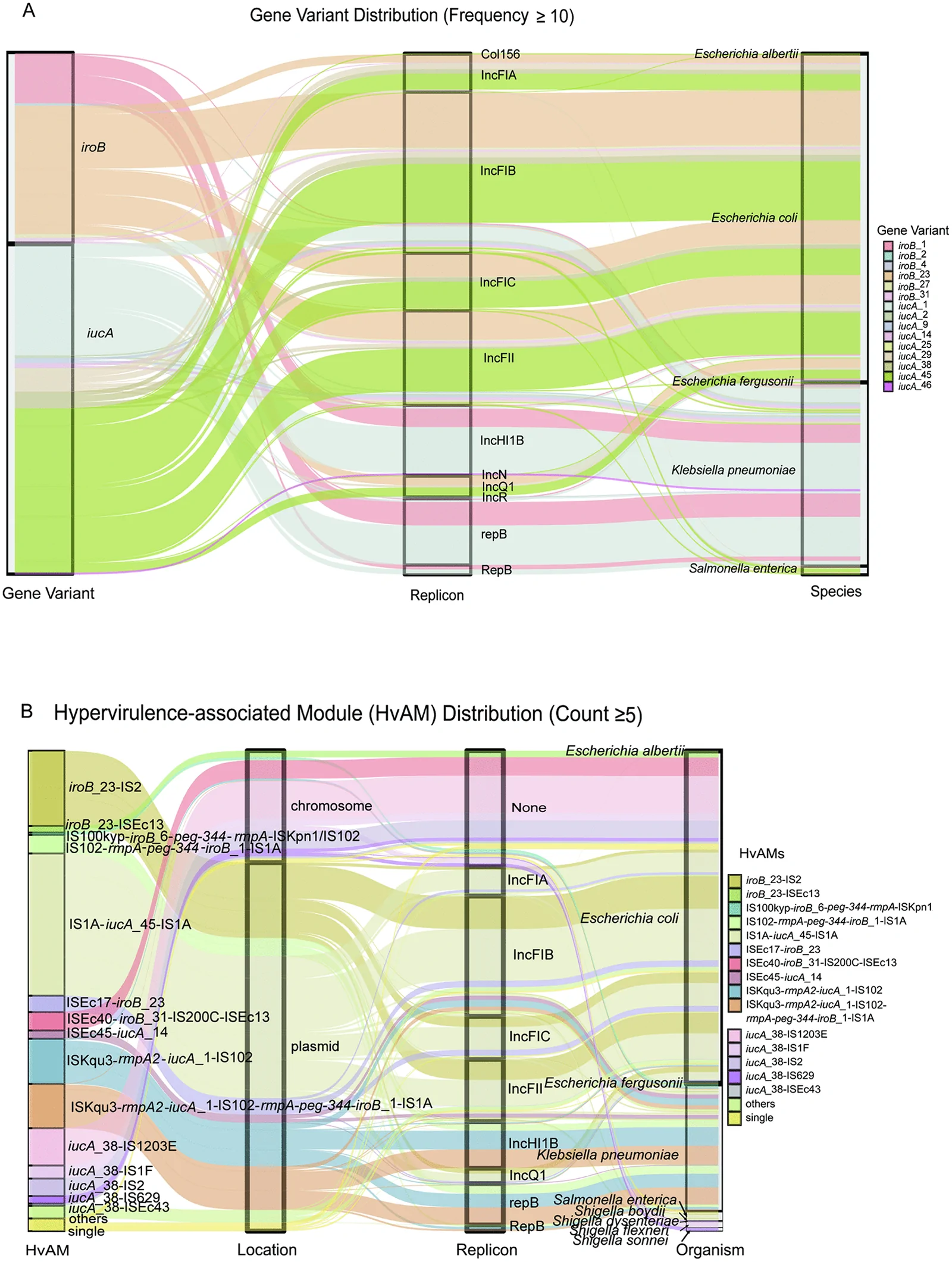
Distribution of hypervirulence-associated gene variants and modules across plasmid replicons and bacterial species. **(A)** Alluvial plot showing the distribution of major *iroB* and *iucA* gene variants (frequency ≥10) across different plasmid replicon types and bacterial species. Flow colours represent different gene variants. **(B)** Alluvial plot displaying the distribution of major Hva-PCTs (count ≥5) across genetic locations (chromosome or plasmid), plasmid replicon types, and bacterial species. Flow colours represent different Hva-PCT.

**Fig 4:**
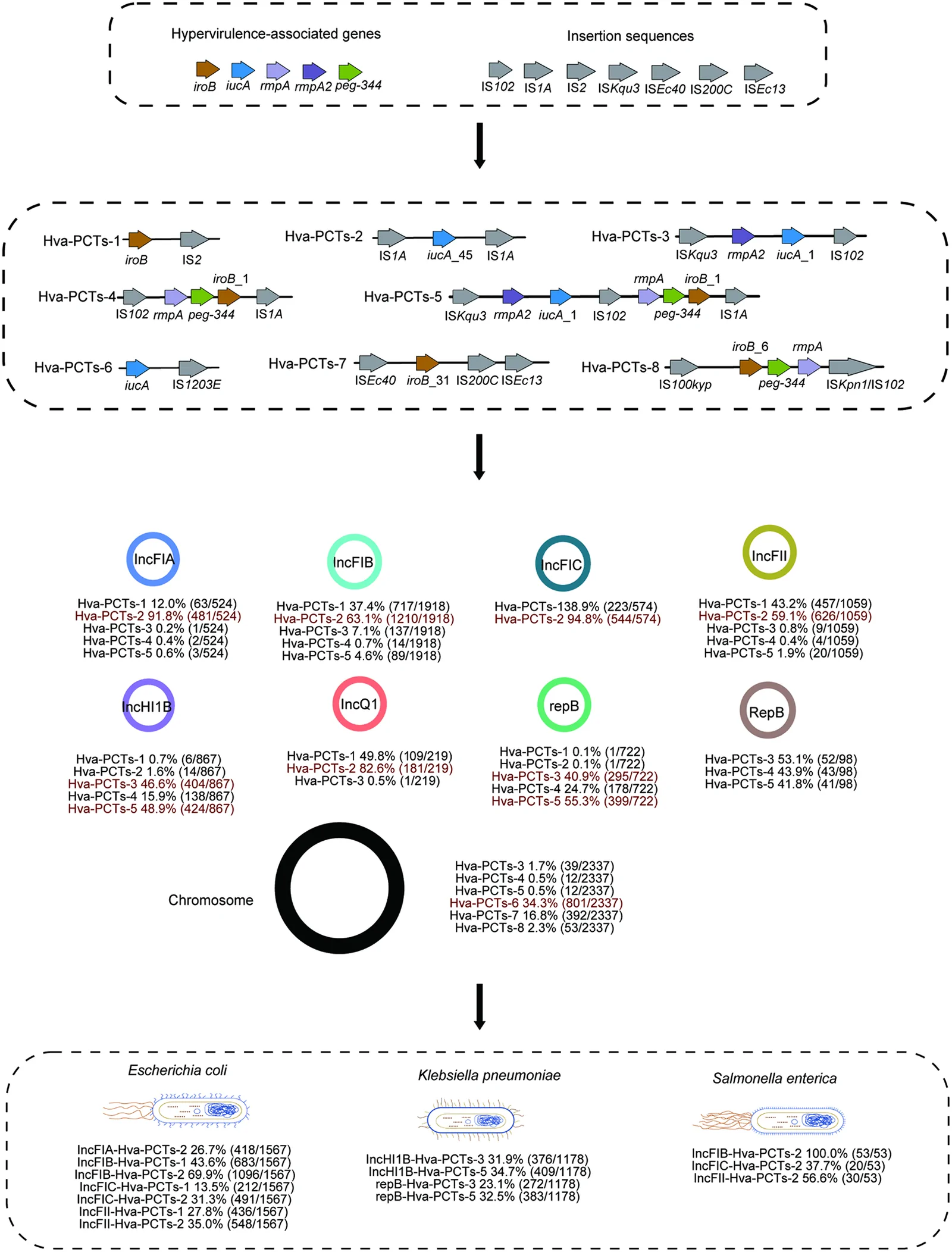
Architecture and distribution of major Hva-PCTs. Schematic diagrams display the structural composition of eight major hypervirulence-associated modules (Hva-PCT-1 to Hva-PCT-8), along with the distribution of these Hva-PCTs across genomic locations and bacterial species.

PHva-PCT distribution was dominated by a handful of variants with strong host and plasmid replicon specificity (Figs 3A, 3B, 4). The two most common—IS*1A‑iucA_45*‑IS*1A* (43.6%, 1,252/2,869) and *iroB_23*‑IS*2* (25.2%, 722/2,869)—together made up 50.9% of all pHva-PCTs (S5 Table). These two structures frequently co-occurred (n = 514) and were found mostly in *E. coli* (n = 482), matching the previously observed co-localization of *iucA_45* and *iroB_2*3 (n = 727) in this species. The most frequent pHva-PCT, IS*1A‑iucA_45*‑IS*1A*, includes the phylogenetically clustered *iucA_29* (n = 138), *iucA_38* (n = 80)*, iucA_46* (n = 6)*, iucA_1* (n = 2) and *iucA_9* (n = 1) variants, and was almost exclusively detected in *E. coli* (88.3%), with low proportions in *K. pneumoniae* (5.8%) and *S. enterica* (4.2%). This variant was overwhelmingly carried by multi-replicon plasmids (n = 1,228); IncFIB was present in 96.6% (n = 1,210) of carriers, followed by IncFII (50.0%) and IncFIC (43.4%) (Fig 3B, S5 Table). Similarly, the second most frequent pHva-PCT, *iroB_23*‑IS*2,* was also carried predominantly by multi-replicon plasmids (n = 694), showed a strong *E. coli* preference (95.2%, 687/722), and was almost always linked to IncFIB (99.3% of carriers), typically in combination with IncFIC(FII) or IncFII. Taken together, these findings indicate that the dissemination of these hypervirulence-associated modules is strongly constrained by both host species and plasmid type. In *E. coli*, multi-replicon plasmids combining IncFIB with IncFIC(FII) and/or IncFII serve as the main vectors. This strong plasmid preference mirrors the natural plasmid repertoires of their hosts: *E. coli* genomes are dominated by multi-replicon plasmids combining IncFIB with IncFIC(FII) and/or IncFII, whereas *K. pneumoniae* carries a higher proportion of IncHI1B- and repB-based multi-Inc-type plasmids (Figs 3B, S5A and S5 Table).

This pattern of strong host-plasmid restriction extended to the remaining pHva-PCTs. Although *iroB* and *iucA* are widespread among Enterobacteriaceae, pHva-PCTs containing specific subtypes of these genes showed a narrow host range; most variants were restricted to a single bacterial host and plasmid replicon type. Among the 30 pHva-PCTs, only two - IS*1R‑iucA_29*‑IS*1R* and IS*1A‑iroB_23* - were found in two host species with comparable frequencies (S5 Table). Even these broader‑host variants exhibited host‑specific plasmid associations. For example, IS*1A‑iroB_23* was detected in *E. coli* (9/19) and *K. pneumoniae* (10/19). In *E. coli*, it was primarily linked to IncFIB/IncFII multi-replicon plasmids; in *K. pneumoniae*, it was associated with IncHI1B/repB multi-replicon plasmids (Fig 3B, S5 Table). Similarly, IS*1R‑iucA_29*‑IS*1R* occured in both *E. coli* (10/18) and *K. pneumoniae* (8/18); it was linked to multi-replicon IncFIB and IncFIA/IncFIC(FII)/IncFII plasmids in *E. coli* but showed multi-replicon IncHI1B/IncFIB linkage in *K. pneumoniae* (Fig 3B, S5 Table).

Among the 30 cHva-PCTs identified on the chromosomes, *iucA_38-*IS*1203E* was the most prevalent (34.3%, 801/2,337) and was almost entirely restricted to *E. coli* (99.6%, 798/801). The most common iroB cHva-PCT, IS*Ec40-iroB_31*-IS*200C* (16.8%, 392/2,337), was found only in *E. coli*. Most cHva-PCTs consisted of either *iucA* or *iroB* individually flanked by IS elements. A notable exception was the multi-gene module IS*100kyp-iroB_6-peg-344-rmpA* (2.3%, 53/2,337), which carries multiple hypervirulence-associated genes and was identified mainly in *K. pneumoniae* (98.1%, 52/53), with a single occurrence in *Klebsiella variicola*.

Three Hva-PCT variants were found on both plasmids and chromosomes; we term these cross-replicon Hva-PCTs (xHva-PCTs) (Fig 2B and 2D). These were predominantly carried by plasmids in *K. pneumoniae* and included: IS*Kqu3‑rmpA2‑iucA_1*‑IS*102* (446/477, 93.5%), IS*Kqu3‑rmpA2‑iucA_1*‑IS*102‑rmpA‑peg‑344‑iroB_1*‑IS*1A* (473/493, 95.9%), and IS*102‑rmpA‑peg‑344‑iroB_1*‑IS*1A* (182/196, 92.9%). The main plasmid replicon types associated with these xHva-PCTs were repB/IncHI1B multi-replicon (Fig 4, S5 Table). These xHva-PCTs were also detected in chromosomal locations, predominantly in *K. pneumoniae* (IS*Kqu3‑rmpA2‑iucA_1*-IS*102*, n = 38; IS*Kqu3‑rmpA2‑iucA_1*‑IS*102‑rmpA‑peg‑344‑iroB_1*‑IS*1A*, n = 12; IS*102‑rmpA‑peg‑344‑iroB_1*-IS*1A*, n = 10). Notably, these composite Hva-PCTs were largely restricted in *K. pneumoniae*, whereas *E. coli* preferentially carried Hva-PCTs consisting of *iucA* or *iroB* singly associated with flanking IS elements, which showed considerable diversity in IS composition (Fig 2F). Interestingly, IS*Kqu3-rmpA2-iucA_1*-IS*102* and IS*102-rmpA-peg-344-iroB_1*-IS*1A* often co-occurred at separate loci on the same plasmid; their physical linkage formed the composite module IS*Kqu3-rmpA2-iucA_1*-IS*102-rmpA-peg-344-iroB_1*-IS*1A*, supporting a stepwise assembly model for virulence acquisition (Fig 2D).

### Cross transmission of Hva-PCTs between plasmids and chromosomes enables both spread and persistence

**Fig 5:**
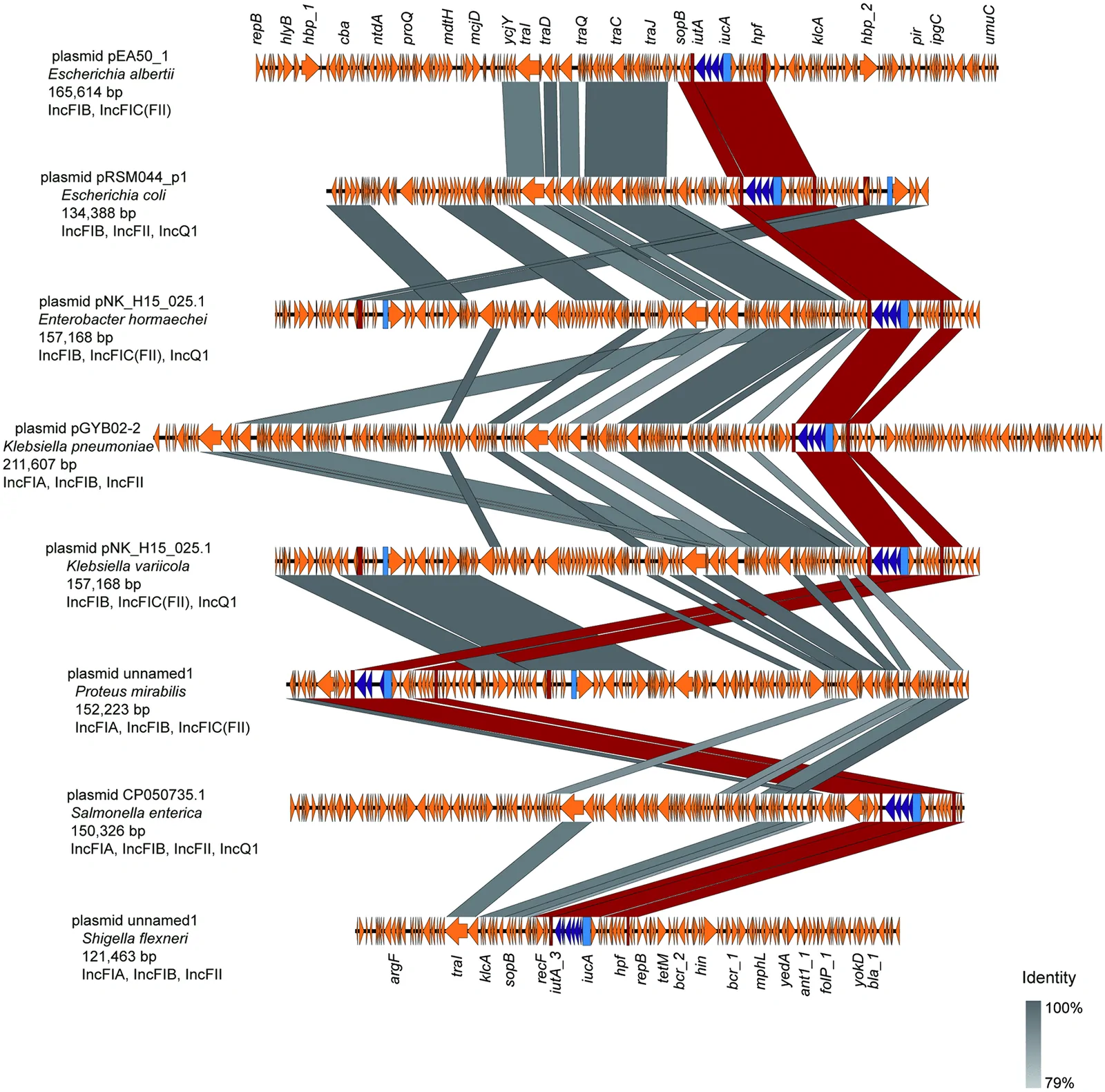
Comparative genomic analysis of the IS*1A-iucA_45-*IS*1A* module across species. In the comparative genomic map, genes are represented as arrows, with their orientation indicating the direction of transcription. Color coding is as follows: orange for other genes, dark red for IS elements within the locus, blue for hypervirulence-associated genes, and purple for *iuc* operon-associated genes. The gray shading between homologous genes denotes regions of collinearity, as identified through whole-genome alignment. The red shading specifically highlights the collinear blocks comprising this conserved hypervirulence locus.

To assess cross-species conservation of the most prevalent Hva-PCTs, we compared representative plasmids from each host species carrying these modules. The dominant pHva-PCT IS*1A*-*iucA*_*45*-IS*1A* was found in seven species: *E. coli, K. pneumoniae, S. enterica, S. flexneri, E. albertii, E. hormaechei, K. variicola,* and *P. mirabilis*. One representative plasmid from each species was selected for pairwise comparison. An approximately 12 kb region containing this pHva-PCT was highly conserved across all eight plasmids, with interspecies pairwise alignments showing average sequence identity of 99.90% ± 0.06% and 100% query coverage (Fig 5). *iucA_45* was the dominant variant, with two exceptions: one P. mirabilis isolate carried *iucA_38*, and the S. enterica plasmid pST90–1 (CP050735) contained *iucA_29*. The overall module structure remained conserved in both cases.

**Fig 6:**
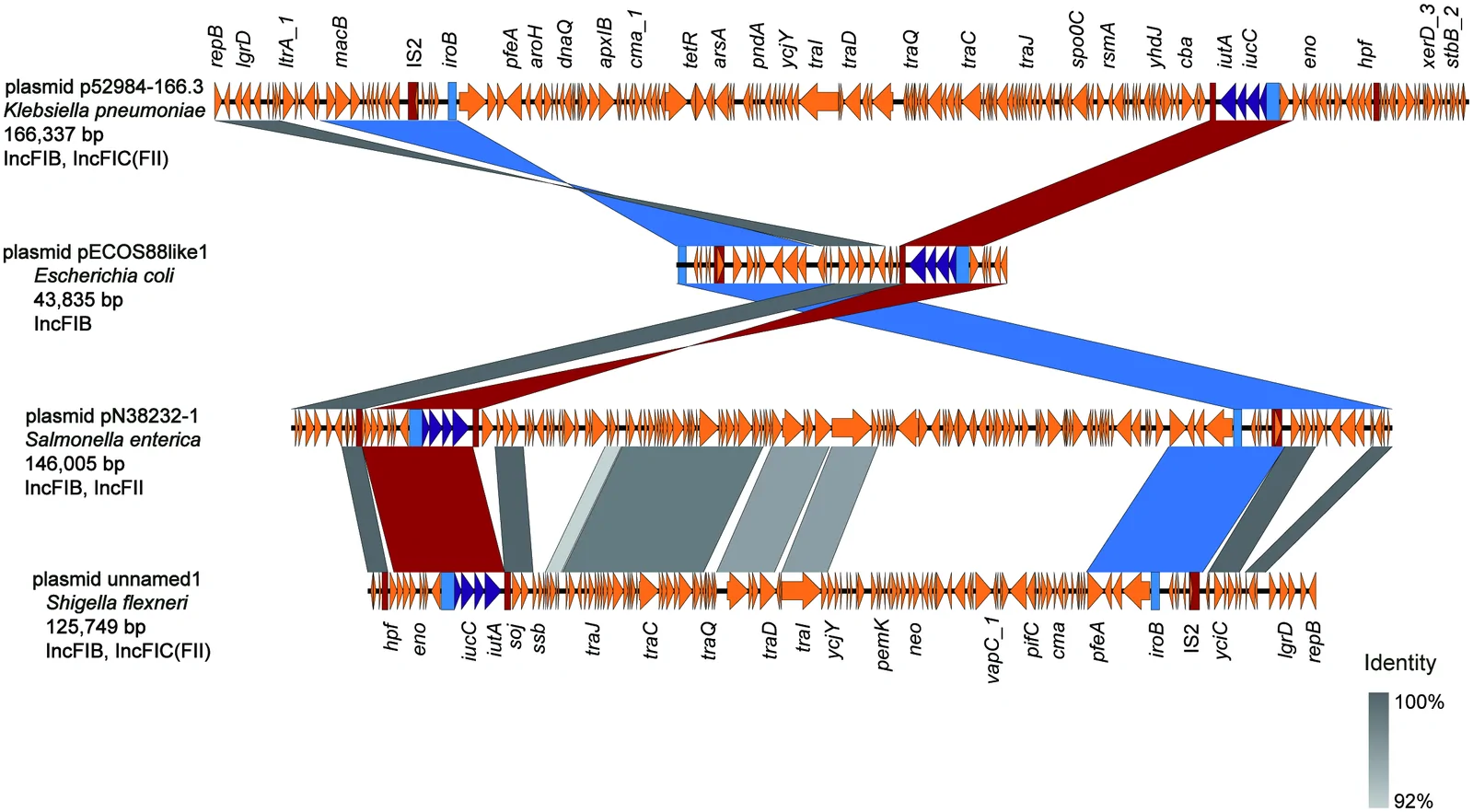
Comparative genomic analysis of the IS*Ec40*-*iroB*_*23/31*-IS*200C*-IS*Ec13* module across species. In the comparative genomic map, genes are represented as arrows, with their orientation indicating the direction of transcription. Color coding is as follows: orange for other genes, dark red for IS elements within the locus, blue for hypervirulence-associated genes, and purple for iuc operon-associated genes. The gray shading between homologous genes denotes regions of collinearity, as identified through whole-genome alignment. The blue shading specifically highlights the collinear blocks comprising IS*Ec40*-*iroB*_*23/31*-IS*200C*-IS*Ec13*, and the red shading highlights the co-occurring IS*1A-iucA_45*-IS*1A* module.

The second most prevalent pHva-PCT, *iroB*_*23*-IS*2*, was also predominant in *E. coli* and co-occurred with IS*1A*-*iucA*_*45*-IS*1A* in most cases (n = 514). We examined representative plasmids from four host species carrying this module - *E. coli, K. pneumoniae, S. enterica*, and *S. flexneri*. Comparative analysis revealed similarly high sequence conservation: an approximately 12 kb region harboring the pHva-PCT showed average nucleotide identity of 99.90 ± 0.06% and 100% coverage across all four species (Fig 6).

**Fig 7:**
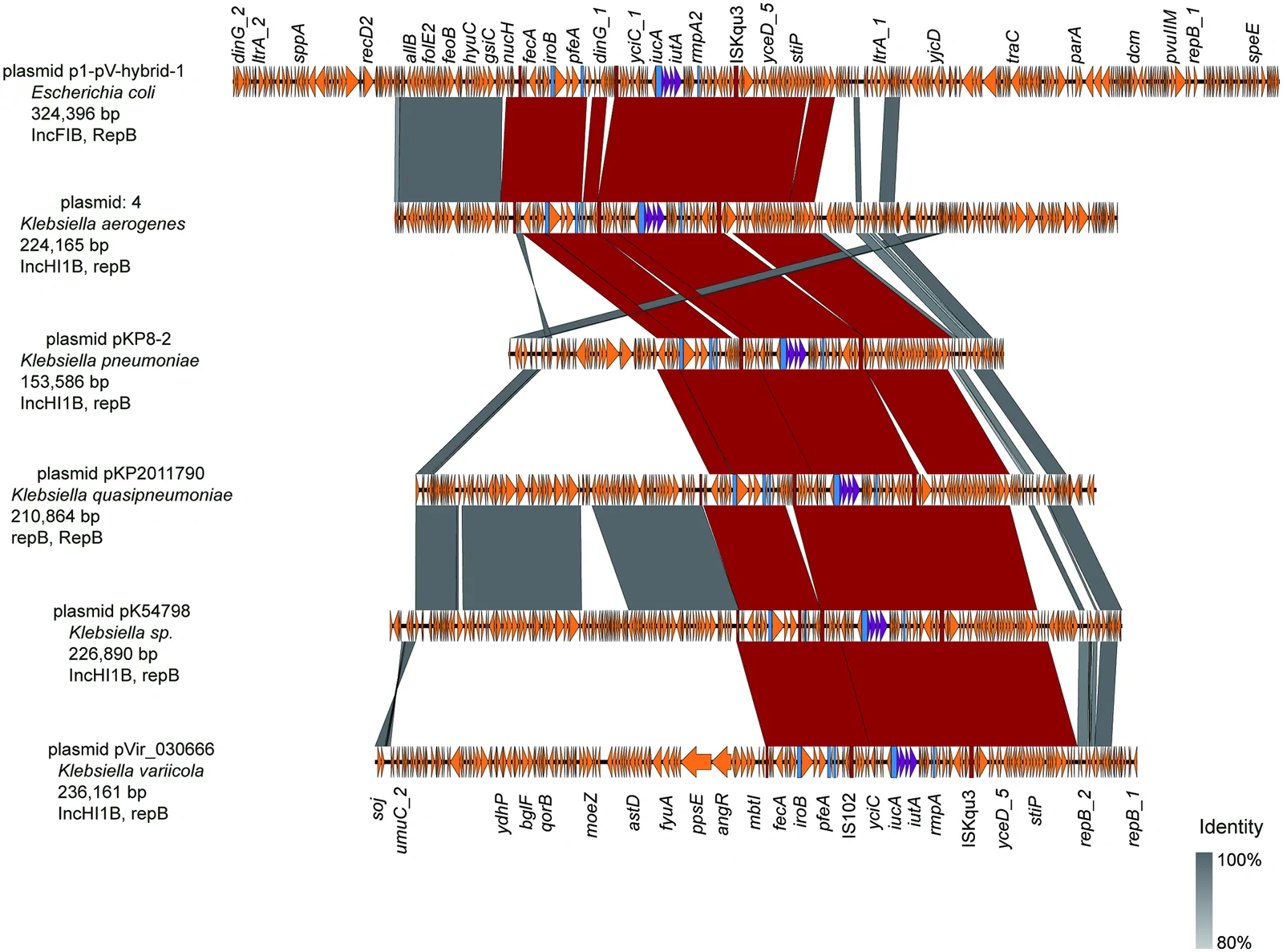
Comparative genomic analysis of the IS*Kqu3*-*rmpA2-iucA*_*1-*IS*102*-*rmpA-peg-344-iroB*_*1*-IS*1A* module across species. In the comparative genomic map, genes are represented as arrows, with their orientation indicating the direction of transcription. Color coding is as follows: orange for other genes, dark red for IS elements within the locus, blue for hypervirulence-associated genes, and purple for iuc operon-associated genes. The gray shading between homologous genes denotes regions of collinearity, as identified through whole-genome alignment. The red shading specifically highlights the collinear blocks comprising this conserved hypervirulence locus.

The three high-frequency xHva-PCTs identified in *K. pneumoniae* showed more complex multi-gene architectures. IS*Kqu3*-*rmpA2*-*iucA_1*-IS*102* and IS*102*-*rmpA-peg-344-iroB_1*-IS*1A* often co-occurred at separate loci on the same plasmid;, their physical linkage formed the composite module IS*Kqu3*-*rmpA2*-*iucA_1*-IS*102*-*rmpA*-*peg-344-iroB_1*-IS*1A*. Comparative analysis of representative strains from all six species carrying this composite module showed >95% sequence similarity across both the xHva-PCT and its flanking regions (Fig 7).

We next examined the chromosomal copies of the three xHva-PCTs. IS*Kqu3*-*rmpA2-iucA_1*-IS*102* was detected exclusively on *K. pneumoniae* chromosomes, whereas IS*102*-*rmpA-peg-344-iroB_1*-IS*1A* and IS*Kqu3*-*rmpA2-iucA_1*-IS*102*-*rmpA-peg-344-iroB_1*-IS*1A* were also found on *K. quasipneumoniae* chromosomes. Pairwise comparison of plasmid and chromosomal copies of each module showed >95% nucleotide identity across all collinear blocks (S6 Fig), indicating that these modules can be stably maintained in both replicon contexts.

In addition to these plasmid-derived xHva-PCTs, we identified a distinct cHva-PCT, IS*100kyp*-*iroB_6-peg-344-rmpA*-IS*Kpn1*/IS*102*. Like the *K. pneumoniae*-predominant xHva-PCTs, this cHva-PCT carried multiple virulence-associated genes and was found almost exclusively on *K. pneumoniae* chromosomes, with a single occurrence in *K. variicola*. Comparative analysis of two *K. pneumoniae* strains (accession number: GCA_002163895.1 and GCA_009497695.1) and one *K. variicola* strain (accession number: GCA_900636185.1) revealed a conserved genomic segment containing this module on their chromosomes (S7 Fig).

### Chromosomal Hva-PCTs exhibit species-specific integration and stable maintenance

We next examined whether cHva-PCTs show sequence conservation consistent with inter-strain transmission. The most prevalent cHva-PCT, *iucA_38*-IS*1203E*, was largely restricted to *E. coli* (794/796, 99.7%), with only one chromosomal occurrence each in *E. hormaechei* and *S. flexneri*. Comparative analysis between the representative *E. coli* strain GCA_002012205.1 and the chromosomes of *E. hormaechei* and *S. flexneri* revealed high sequence similarity and coverage across the module-flanking region (*E. hormaechei*: 97.72% identity, 89.11% coverage; *S. flexneri*: 97.71% identity, 89.13% coverage) (S8 Fig). Similarly, IS*Ec40*-*iroB_23*-IS*200C*-IS*Ec13*, another recurrent cHva-PCT, was exclusively in *E. coli* and co-occured with *iucA_38*-IS*1203E* in a subset of strains. Comparative analysis confirmed that this module was also stably maintained on the *E. coli* chromosome with high sequence conservation (identity: 98.17%; coverage: 93.14%) (S9 Fig).

A distinct cHva-PCTs, IS100kyp-*iroB_6-peg-344-rmpA*-ISKpn1/IS102, was identified primarily on the *K. pneumoniae* chromosome. Comparative analysis of two *K. pneumoniae* chromosomes (GCA_002163895.1, GCA_009497695.1) and one *K. variicola* chromosome (GCA_900636185.1) revealed a conserved genomic segment containing this module across all three chromosomes (S7 Fig). Unlike the plasmid-derived xHva-PCTs, this cHva-PCT typically occurred independently of other hypervirulence modules.

For the three xHva-PCTs found on both plasmids and chromosomes, comparative analysis confirmed that their plasmid and chromosomal copies share extensive collinear blocks without major structural variations, indicating stable maintenance in both replicon contexts (S6 Fig). The submodules IS*Kqu3*-*rmpA2-iucA_1*-IS*102*, and IS*102*-*rmpA-peg-344-iroB_1*-IS*1A* were capable of independent dissemination, and their physical linkage formed the composite IS*Kqu3*-*rmpA2-iucA_1*-IS*102*-*rmpA-peg-344-iroB_1*-IS*1A* module. Notably, one *K. pneumoniae* strain (GCA_002870865.1) carried an identical copy of this composite module on both its chromosome and a resident plasmid (S6 Fig), providing direct evidence that the same Hva-PCT can be maintained simultaneously in both genomic compartments within a single strain.

**Fig 8:**
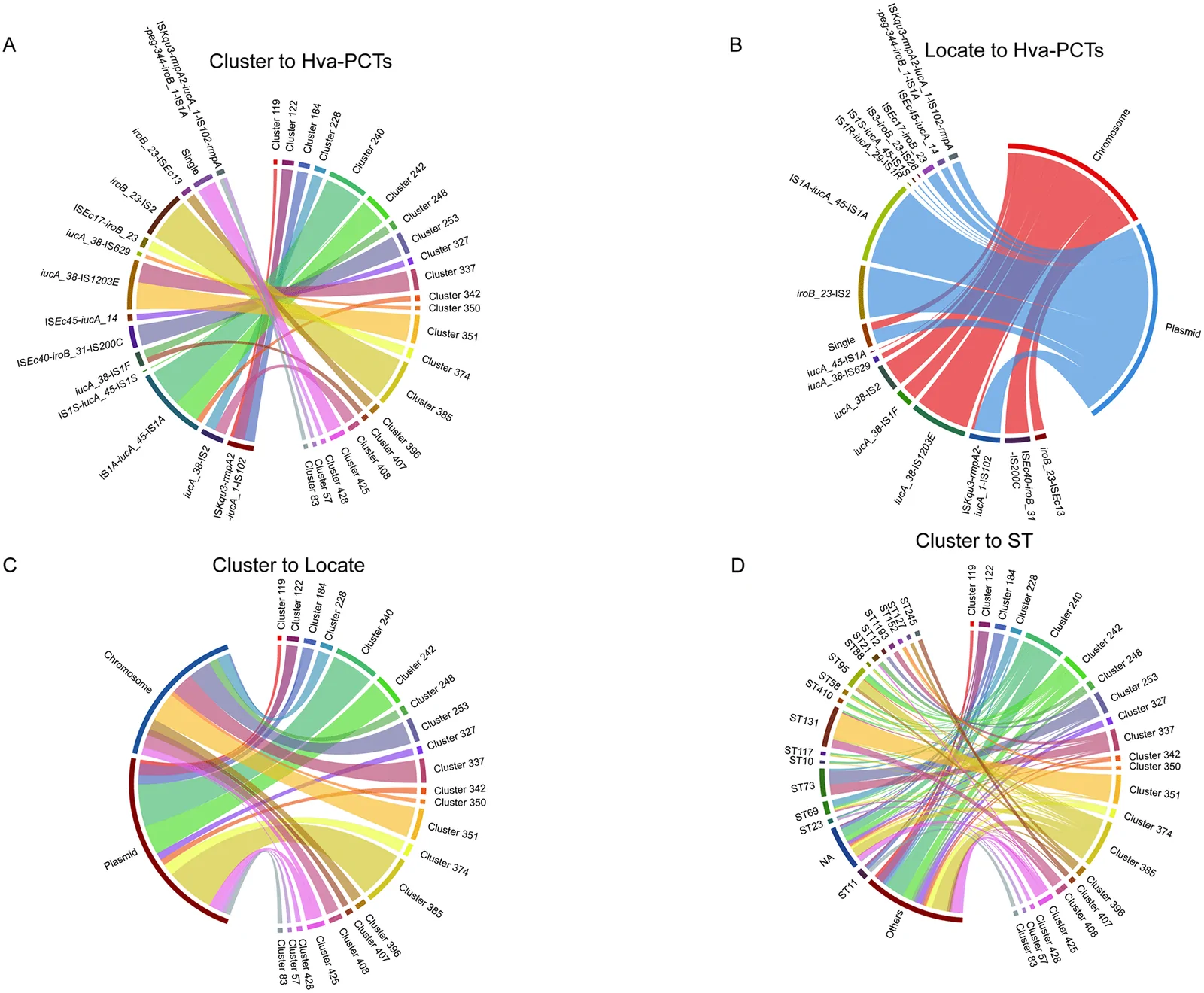
Chord diagrams showing flow distributions between different classification levels. (A) Flow relationships from Cluster to Hva-PCTs; (B) Flow relationships from locate to Hva-PCTs; (C) Flow relationships from Cluster to locate; (D) Flow relationships from Cluster to ST. Only Clusters with frequency ≥ 50 were retained. ST types with frequency < 50 were grouped as “Others.” Hva-PCTs were not filtered and kept all original types. For chord visualization, only connections with flow intensity ≥ 10 are shown. The width of each chord represents the flow intensity. Different sectors are distinguished by distinct colors, with labels arranged around the circumference. These diagrams illustrate the flow patterns among major taxonomic units, revealing hierarchical associations from Cluster to Hva-PCTs, locate, and ST.

To obtain a global, sequence-based assessment of the relationship between plasmid-borne and chromosomal Hva-PCTs, we pooled all extracted Hva-PCT sequences (plasmid and chromosomal copies combined) and performed CD-HIT clustering at 80% nucleotide identity and 90% coverage. This analysis identified 433 clusters in total, of which 22 contained fifty or more members. Of these 22, 13 clusters (59.1%) included both plasmid and chromosomal copies, indicating that most major Hva-PCT sequence clusters are shared across the two replicon types (S6 Table). The mean intra-cluster nucleotide identity exceeded 80% for all shared clusters. Furthermore, these shared clusters were not confined to a single ST or species: most contained representatives from multiple STs, and several spanned two or more host species (S6 Table). To visualize the hierarchical relationships among these clusters, we constructed chord diagrams showing flow patterns between clusters and Hva-PCTs (Fig 8A), between genomic location (plasmid/chromosome) and Hva-PCTs (Fig 8B), between clusters and genomic location (Fig 8C), and between clusters and STs (Fig 8D). Collectively, these clustering results and flow visualizations indicate that conserved Hva-PCT modules circulate broadly across plasmid and chromosomal compartments, as well as across strain and species boundaries, rather than being restricted to clonal lineages or a single replicon type.

### Gene amplification within Hva-PCTs underscores their evolutionary plasticity and potential for enhanced virulence

During our analysis of hypervirulence-associated genes, we identified several instances of gene multicopy amplification. One example is plasmid pP901 (Accession number: CP050247.1) (S10B Fig), which carries multiple copies of *rmpA2* and *iucA_1*. This plasmid was isolated from a human clinical sample in China (2018) and belongs to Clade 2 of the *iucA* phylogenetic tree and originates from *K. pneumoniae* (S3A Fig). While a reference pLVPK-like plasmid contains the complete five-gene set arranged as IS*Kqu3-rmpA2-iucA_1*-IS*102-rmpA-peg-344-iroB_1-*IS*1A*, plasmid pP901 exhibits a partial duplication at both termini. The resulting structure, IS*Kqu3-rmpA2-iucA_1-*IS*102* (which lacks the terminal IS*102* element in the duplicated segment), is consistent with our proposed model how complete hypervirulence gene clusters disseminate.

## Discussion

The chromosomal integration of virulence genes has emerged as a key evolutionary mechanism in the rise of CR-hvKP. Early evidence came from a detailed analysis of the ST11 CR-hvKP clinical isolate 16HN-263, where a virulence segment carrying *rmpA2* and *iucABCD-iutA* was found flanked by IS*26* elements on the chromosome. The same study showed that this fragment could form a circular intermediate, pointing to IS*26*-mediated chromosomal integration as a clinically relevant route for virulence module dissemination [16]. Later large-scale genomic work on the ST11-KL47 lineage revealed that the core virulence module (*rmpA2* and *iucABCD-iutA*) can become permanently fixed at a specific chromosomal locus, with homologous recombination likely initiated by mobile elements such as IS*Kpn28* and IS*26* originating from fused plasmids [44]. Together, these studies established IS-orchestrated chromosomal integration as a widespread and stable strategy in CR-hvKP evolution. However, they focused mainly on a few high-risk *K. pneumoniae* clones. Whether IS-mediated mobility of virulence-associated modules is a general phenomenon across the broader Enterobacteriaceae, and what structural forms these modules take in other species and genomic contexts, have remained open questions. Our study begins to address these gaps.

We present here a module-centered view of how hypervirulence-associated genes disseminate across Enterobacteriaceae. Rather than treating complete virulence plasmids as the primary vehicles of transmission, our data suggest that smaller IS-bounded modules represent more fundamental and traceable units of mobility. Virulence plasmids are often structurally dynamic, with frequent recombination, insertions, deletions, and backbone replacements. Under such conditions, plasmid-level comparisons alone can easily obscure the conserved mobile components that actually drive the movement of virulence determinants. By focusing on Hva-PCTs, we shift the analytical framework from “which plasmid carries virulence genes” to “which mobile module is being transferred, remodeled, and maintained.” One illustrative example is *K. pneumoniae* GCA_002870865.1, which carries an identical composite Hva-PCT on both its chromosome and a plasmid—a pattern that would be missed by plasmid-centric analysis alone.

This module-level perspective helps explain why hypervirulence-associated loci are found across diverse genomic backgrounds. We identified multiple Hva-PCTs that exist in near-identical form (>95% nucleotide identity) in different host species. The dominant IS*1A-iucA_45*-IS*1A* module, for instance, was detected in eight species spanning *Escherichia*, *Klebsiella*, *Salmonella*, and *Enterobacter*, with interspecies pairwise alignments averaging 99.9% identity. CD-HIT clustering further showed that 59.1% of major Hva-PCT sequence clusters contain both plasmid and chromosomal representatives, spanning multiple sequence types and host species. Thus, the spread of hypervirulence appears to be driven not solely by clonal expansion or intact plasmid circulation, but by these IS-linked modules that can escape their original contexts and establish themselves in new plasmid or chromosomal environments.

To ground our structural findings in a clinically relevant context, we examined the PCT architecture in the ST23-KL1 lineage of *K. pneumoniae*, the dominant hypervirulent clone associated with pLVPK-like virulence plasmids. Using AY378100.1 as a reference, we searched ST23-KL1 genomes with thresholds of 70% coverage and 80% identity. A total of 35 pLVPK-like virulence plasmids were identified in this lineage. Of these, 32 (91.4%) carried the complete IS*Kqu3-rmpA2-iucA_1*-IS*102-rmpA-peg-344-iroB_1*-IS*1A* module (the full pHva-PCT configuration); three carried a partial configuration consisting of either IS*102-rmpA-peg-344-iroB_1*-IS*1A* or IS*Kqu3-rmpA2-iucA_1*-IS*102*; the remaining two showed other variant structures (S7 Table). These results indicate that the complete pHva-PCT architecture is highly prevalent among pLVPK-like virulence plasmids in ST23-KL1, while partial configurations occur at lower frequency, consistent with either stepwise assembly or gradual decay of the module. This model is further supported by recent experimental evidence from Sun et al., who demonstrated that IncFIB(Mar) plasmids play a central role in the emergence of hypervirulent carbapenem-resistant *K. pneumoniae* by serving as conjugative vehicles for the dissemination of acquired virulence-associated regions [28]. These findings provide independent evidence that IncFIB(Mar)-related plasmids can acquire and disseminate conserved virulence-associated regions, supporting the broader plausibility of module-level virulence-gene mobility.

The observed associations between specific Hva-PCT categories and particular bacterial hosts or plasmid backbones suggest that ecological and genetic compatibility shape the dissemination of hypervirulence. Siderophore-associated modules, such as those carrying aerobactin or salmochelin loci, may have broader functional portability because iron acquisition is advantageous across many Enterobacteriaceae under host-imposed nutritional immunity [5, 7, 45]. In contrast, regulators like *rmpA* and *rmpA2* may depend more on the recipient's capsule biosynthesis background and regulatory architecture [5, 32]. This difference likely explains why distinct virulence modules show different host distributions and plasmid associations. Successful spread of a virulence module therefore depends not only on its mobility but also on whether it can be functionally accommodated by the recipient genome.

The strong host specificity of Hva-PCTs raises the question of their biological and clinical relevance in non-*Klebsiella* Enterobacteriaceae. Our data show that *iucA/iro*B-type modules are overwhelmingly associated with *E. col*i and its characteristic IncFIB-based multi-replicon plasmids, suggesting that *E. coli* may act as a mobilization-competent reservoir for these siderophore-encoding modules. While cross-species transfer to *Klebsiell*a appears limited under current conditions, the barrier is not absolute: several Hva-PCT sequence clusters span multiple host species (S6 Table), and the three xHva-PCTs were detected in both *K. pneumoniae* and *K. quasipneumoniae*. The species barrier could potentially be breached through acquisition of compatible conjugative machinery or under appropriate selective pressure. Clinically, although the classical hypervirulence phenotype is defined primarily in *K. pneumoniae*, the presence of iuc/iro-type Hva-PCTs in *E. coli*, *Salmonella enterica*, and *Shigella* species may confer fitness advantages not captured by current virulence definitions. Siderophore-mediated iron acquisition is already recognized as a virulence mechanism in extraintestinal pathogenic *E. coli* [45], and the chromosomal integration of these modules in certain *E. coli* lineages suggests long-term adaptive value. Monitoring Hva-PCTs in non-*Klebsiella* hosts is therefore relevant not only for tracking potential reservoirs of mobile virulence elements but also for understanding how these modules contribute to pathogenesis in their native host backgrounds.

Our findings also suggest that the boundary between plasmid-mediated and chromosome-mediated virulence inheritance may be more fluid than previously thought. Plasmids are typically viewed as drivers of rapid horizontal gene transfer, while chromosomes are seen as stable platforms for vertical inheritance [46, 47]. The presence of highly similar Hva-PCT structures in both replicon types indicates that these two routes are not independent. Instead, plasmids and chromosomes may form a connected evolutionary network through which virulence modules circulate, integrate, and persist. In this model, plasmids facilitate rapid dissemination, whereas chromosomal integration may stabilize virulence determinants once they enter a suitable host background, potentially accelerating the long-term fixation of hypervirulence traits in clinically important lineages.

The stepwise assembly of complex Hva-PCTs further supports the idea that hypervirulence evolves through modular accretion rather than by acquisition of a single complete virulence region. Simple IS-linked units may recombine with each other or insert into pre-existing virulence-associated regions, gradually building larger and more complex cassettes. This process parallels the modular evolution well documented in antimicrobial resistance regions, where insertion sequences and transposon-like structures assemble multiple resistance genes into transferable clusters [9, 48]. Our findings suggest that a similar principle operates in virulence evolution. However, unlike resistance genes, the phenotypic effects of virulence genes are often more context-dependent; acquiring a module does not guarantee a hypervirulent phenotype. Functional validation remains essential to determine how different Hva-PCTs contribute to pathogenicity in different host backgrounds. Our observation that IS*Kqu3-rmpA2-iucA_1*-IS*102* and IS*102-rmpA-peg-344-iroB_1*-IS*1A* frequently co-occur as separate entities on the same *K. pneumoniae* plasmid, and that their collinear arrangement generates the full composite module, directly supports this model. The partial duplication event in plasmid pP901 further illustrates how additional structural diversification can occur after initial assembly.

From an epidemiological perspective, Hva-PCTs may serve as useful units for genomic surveillance. Current surveillance strategies often focus on species identification, sequence type, resistance genes, virulence genes, or plasmid replicon types [49, 50]. While informative, these markers do not fully capture the mobility potential of virulence determinants. Tracking Hva-PCT structures could help determine whether virulence genes are embedded in mobile contexts and whether similar modules are circulating across species, plasmids, or chromosomes. This approach may improve risk assessment by distinguishing incidental carriage of virulence genes from carriage of potentially transferable virulence modules—a distinction that is particularly relevant for monitoring the emergence of strains that combine hypervirulence with antimicrobial resistance.

Several limitations of this study should be acknowledged. First, the mobility of Hva-PCTs was inferred primarily from comparative genomic evidence. Although conserved IS-flanked structures and their presence across different genetic contexts strongly suggest mobility, direct experimental assays are needed to demonstrate transposition, recombination, or inter-replicon transfer. Second, directionality of transfer between species, plasmids, and chromosomes cannot be determined from genomic snapshots alone. Associations with specific hosts or plasmid types should therefore be interpreted as evolutionary signals rather than definitive evidence of origin. Third, the biological contribution of different Hva-PCTs to virulence was not experimentally assessed. Because virulence expression depends on recipient background, regulatory compatibility, capsule type, and host environment, future studies should combine comparative genomics with phenotypic validation and infection models.

## Conclusion

In conclusion, our findings support a model in which hypervirulence-associated genes in Enterobacteriaceae disseminate through IS-associated modular units rather than solely through intact virulence plasmids. Hva-PCTs provide a mechanistic and surveillance-relevant framework for understanding how virulence determinants are assembled, transferred, and stabilized across bacterial populations. Recognizing these modules as functional risk units may improve genomic monitoring of hypervirulent pathogens and help anticipate the emergence of high-risk strains carrying both virulence and antimicrobial resistance determinants.

## Supporting information

S1 Table Species distribution and genome counts of the 19,601 Enterobacteriaceae genomes included in this study. (XLSX)

S2 Table List of 4,491 Enterobacteriaceae genomes carrying at least one hypervirulence-associated gene (*iucA, iroB, rmpA, rmpA2*, and/or *peg-344*), showing genomic location (chromosome or plasmid) and gene variant for each detected locus. (XLSX)

S3 Table Co-occurrence patterns of the five hypervirulence-associated genes across genomic contexts. Sheet 1: co-occurrence across all genomic locations; Sheet 2: chromosomal co-occurrence matrix; Sheet 3: plasmid co-occurrence matrix.
(XLSX)

S4 Table Complete list of Hva-PCTs identified in this study. Sheet 1: 30 chromosomal Hva-PCTs (cHva-PCTs); Sheet 2: 29 plasmid-borne Hva-PCTs (pHva-PCTs); Sheet 3: distribution of each pHva-PCT across multi-replicon, single-replicon, and no-replicon plasmids.
(XLSX)

S5 Table Matrix showing the distribution of 2,869 virulence-associated plasmids by pHva-PCT type and plasmid replicon type(s). (XLSX)

S6 Table CD-HIT clustering results of Hva-PCT sequences at 80% nucleotide identity and 90% coverage, showing cluster composition and distribution across replicon types, sequence types (STs), and host species. (XLSX)

S7 Table Prevalence of complete and partial Hva-PCT configurations among 35 pLVPK-like virulence plasmids identified in the ST23-KL1 lineage of *K. pneumoniae.* (XLSX)

S1 Fig Heatmap illustrating the distribution of major *iroB* and *iucA* gene variants across species, without distinguishing genetic location. Colour intensity represents the relative abundance of each variant within a given species.
(TIF)

S2 Fig Species-specific distribution and genetic localization of hypervirulence-associated genes and their major variants. (A) Heatmap displaying the distribution and genetic location of the five hypervirulence-associated genes across Enterobacteriaceae species. For each gene–species pair, cell colour indicates presence rate; the accompanying text shows the absolute number of isolates and the percentage located on chromosome or plasmid. (B-E) Paired heatmaps detailing the distribution of major *iroB* and *iucA* variants, stratified by genetic location (top: chromosomal; bottom: plasmid).
(TIF)

S3 Fig Phylogenetic analysis of *iucA* and *iroB* genes. (A) Circular phylogenetic tree of *iucA*, with layers from inner to outer representing species, *iucA* variants, replicon types, and Hva-PCTs. (B) Circular phylogenetic tree of *iroB* with corresponding layers. (C) Unrooted phylogenetic tree of *iroB* gene sequences showing major clades. (D) Unrooted phylogenetic tree of *iucA* gene sequences displaying clade structure and phylogenetic topology.
(TIF)

S4 Fig Phylogenetic analysis of *rmpA, rmpA2*, and *peg-344* genes. (A-C) Circular phylogenetic trees with concentric rings showing (from inner to outer): species distribution, plasmid replicon types, and Hva-PCTs.
(TIF)

S5 Fig Plasmid replicon type distribution and host species associations. (A) Distribution of Inc types across species. (B) Proportional distribution of plasmid replicon types identified from 2,869 virulence-associated plasmids (low-frequency types, count <10, grouped as “Others”). Surrounding bar plots detail host species composition for the six most prevalent replicon types (IncFIB, IncFII, IncHI1B, repB, IncFIC, and IncFIA).
(TIF)

S6 Fig Comparative genomic analysis of the IS*Kqu3-rmpA2-iucA_1*-IS*102-rmpA-peg-344-iroB_1*-IS*1A* module across species. Arrows indicate genes and their transcriptional orientation. Colour coding: orange, other genes; green-blue, IS elements; red, hypervirulence-associated genes. Grey shading denotes collinear regions identified by whole-genome alignment.
(TIF)

S7 Fig Comparative genomic analysis of the IS*100kyp-iroB_6-peg-344-rmpA*-IS*Kpn1*/IS*102* module across species. Colour coding: orange, other genes; green-blue, IS elements; red, hypervirulence-associated genes. Grey shading denotes collinear regions.
(TIF)

S8 Fig Comparative genomic analysis of the *iucA_38*-IS*1203E* module across species. Colour coding: orange, other genes; green-blue, IS elements; red, hypervirulence-associated genes. Grey shading denotes collinear regions; red shading highlights the conserved hypervirulence locus.
(TIF)

S9 Fig Comparative genomic analysis of the IS*Ec40-iroB_31*-IS*200C*-IS*Ec13* module across species. Colour coding: orange, other genes; green-blue, IS elements; red, hypervirulence-associated genes. Grey shading denotes collinear regions; red shading highlights the conserved hypervirulence-associated locus.
(TIF)

S10 Fig Synteny comparisons illustrating gene amplification events. (A) Alignment between the multi-copy *iroB* plasmid CP032832.1 and a canonical single-copy *iroB* virulence-associated plasmid. Hypervirulence-associated genes are shown in red; IS elements in blue. The absence of flanking IS elements for *iroB* copies in CP032832.1 argues against canonical Hva-PCT formation. (B) Alignment between the multi-copy *rmpA2/iucA_1* plasmid GCA_044793255.1_CP050247.1 and a canonical virulence plasmid, consistent with tandem duplication of the entire hypervirulence-associated module.
(TIF)

S1 Appendix This appendix provides detailed phylogenetic and genomic analyses of hypervirulence-associated genes and mobile modules as a supplement to the main text. (DOCX)
