# Supplementary material for: Hypervirulence-associated pseudo-compound transposons as fundamental mobile units driving cross-species virulence dissemination in Enterobacteriaceae
Source: PLoS Pathog. 2026 Sep 1;22(9):e1014513. doi: 10.1371/journal.ppat.1014513 (PMC13533425; doi:10.1371/journal.ppat.1014513)
Supplement: Supplementary file 1 [file ppat.1014513.s018.docx]

**Supplementary Appendix**

**Supplementary Result**

**1.Phylogenetic Analysis of Hypervirulence-Associated Genes**

To comprehensively analyze the distribution and transmission of hypervirulence-associated genes, we also retrieved all 135,570 plasmid sequences from NCBI and performed a genome-wide screen, and identified 2,697 virulence-associated plasmids carrying at least one of the five genes (*iroB, iucA, rmpA, rmpA2,* and *peg-344*). After removing the duplicates with those from the complete genomes in above analyses, a total of 2,869 virulence-associated plasmids were retained. From the 2,337 chromosomal and 2,869 plasmid sequences carrying hypervirulence-associated genes, the sequences of these genes were extracted and used to construct maximum-likelihood trees (S3, S4 Fig). Phylogenetic analysis revealed distinct evolutionary patterns: *iroB* and *iucA* exhibited broad host distribution and high variant diversity, whereas *rmpA*, *rmpA2*, and *peg-344* demonstrated pronounced species specificity towards *K. pneumoniae*.

The *iroB* genes were clustered into three major groups, clade 1-3 (Supplementary Figure S3A, S3C). Clade 1 and 2 were predominantly from *E. coli*, while clade 3 primarily from *K. pneumoniae*. Clade 1 contained 421 *iroB* sequences (421/1,672, 25.2%), overwhelmingly from *E. coli* (420/421, 99.7%), and only one sequence were from a *K. pneumoniae* plasmid. Meanwhile, the majority of clade 1 were located in chromosomes (91.4%). Two *iroB* variants were identified in clade 1, and *iroB*_*31* was the dominant (92.4%), followed by *iroB*_*23* (7.6%). Both variants were primarily chromosomally encoded (*iroB*_*31*: 99.0%; *iroB*_*23*: 90.6%). Norably, the chromosomal *iroB*_*31* variants in clade 1 frequently co-occurred with the *iucA*_*38* variant. Clade 2 comprised 822 *iroB* sequences (822/1,672, 49.2%), also predominantly originating from *E. coli* (780/822, 94.9%). Interestingly, this clade was dominantly plasmid-borne (90.4%). Two *iroB* variants were identified, dominated by the *iroB*_*23* variant (96.7%), with a minor of *iroB*_*27* (3.3%). On plasmids, *iroB*_*23* showed a strong associated with *iucA*_*45*, co-occurring in 486 out of 679 cases (71.6%). Clade 3 comprised 429 *iroB* sequences (429/1,672, 25.7%), predominantly originated from *K. pneumoniae* (94.9%). The majority of this clade were plasmid-borne (85.1%). This clade comprised of 11 variants, dominated by *iroB*_*1*variant (80.9%) and *iroB*_*6* (7.2%)variant. The *iroB*_*1* variant was primarily plasmid encoded (93.7%), whereas *iroB*_*6* was exclusively chromosomally-located. Strikingly, nearly all chromosomal *iroB* sequences in Clade 3 co-occurred with both *peg-344* and *rmpA*. Furthermore, the plasmid-borne *iroB*_*1* variant in Clade 3 almost invariably co-occurred with the complete set of *iucA*_*1*, *peg-344*, *rmpA*, and *rmpA2*, a co-localization pattern that was absent from Clades 1 and 2.

The *iucA* genes were also clustered into three major groups, clade 1-3 (S3B Fig). Another 23 isolates formed separate singleton branches and could not be assigned to any major clade. Clade 1 contained 1,053 *iucA* sequences (1,053/3,438, 30.6%), dominantly originated from *E. coli* (937/1,053, 89.0%) and plasmid-borne (94.8%). This clade comprised the *iucA*_*45* (82.3%), *iucA*_*29* (11.2%) and *iucA*_*38* (2.7%) variants, with *iucA*_*45* frequently co-occurring with *iroB*_*23*. Clade 2 comprised of 903 sequences (903/3,438, 26.3%), predominantly originating from *K. pneumoniae* (91.1%) and plasmid-borne (89.3%). This clade contained 21 *iucA* variants, and was dominated by the *iucA*_*1* variant (78.7%), which consistently co-occurred *with* iroB_*1*. Clade 3 comprised of 1,459 *iucA* sequences (1,459/3,438, 42.4%), predominantly originating from *E. coli* (1,256/1,459, 86.1%) and chromosomally encoded (1,399/1,459, 95.9%). All *iucA* sequences in this clade belonged to the *iucA*_*38* variant, which was frequently found to co-occur with the *iroB*_*31* variant.

In contrast to *iroB* and *iucA*, which exhibit broader taxonomic distributions, over 90% of *rmpA*, *rmpA2,* and *peg-344* occurrences were confined to *K. pneumoniae*. Given this strong species specificity, we therefore selected isolates of these three genes to investigate their phylogenetic structure and cross-species transmission dynamics (S4 Fig). Phylogenetic analysis revealed a consistent pattern across all these three genes. The 785 *rmpA* sequences were from 6 species, consisted primarily of *K. pneumoniae* (95.4%), with the vast majority of sequences being plasmid-borne (89.3%) (S2A Fig). Similarly, the 1,009 *rmpA2* sequences from 8 species was dominated by *K. pneumoniae* (95.1%) and exhibited an even higher prevalence of plasmid carriage (94.8%) (S2A Fig). The 792 *peg-344* sequences from 6 species also showed a strong species specificity to *K. pneumoniae* (95.5%), with most genes located on plasmids (89.1%) (S2A Fig). Although predominantly restricted to *K. pneumoniae*, low-frequent occurrences of these genes were also detected in other species, including *E. coli, E. hormaechei, K. quasipneumoniae, K. variicola, Klebsiella sp.*, and *K. aerogenes*. In addition, the genes identified in other speices were clustered together with those from *K. pneumoniae*, indicating cross-species transmission. We then analyzed and explored the evolutionary associations and potential co-transmission mechanisms of these hypervirulence associated genes and variants in the following section.

**2.Cross-species horizontal transmission of high-frequency Hva-PCTs via plasmids**

The IS*1A*-*iucA*_*45*-IS*1A* module was identified in seven species: *E. coli* (1,06/1,252, 88.3%)*, K. pneumoniae* (73/1,252, 5.8%)*, Salmonella enterica* (53/1,252, 4.2%)*, Shigella flexneri* (9/1,252, 0.7%)*, Escherichia albertii* (6/1,252, 0.5%)*, Enterobacter hormaechei* (1/1,252, 0.08%)*, Klebsiella variicola* (1/1,252, 0.08%)*, Proteus mirabilis* (1/1,252, 0.08%). Recombination analysis revealed that representative strains from these species shared nearly identical sequences within this hypervirulence module (Fig 5). Although the *iucA*_*45* was the dominate variant, exceptions were observed: one *P. mirabilis* isolate carrying *iucA*_*38* within this module, and the plasmid pST90-1 from *S. enterica* strain ST90 (accession number: CP050735) contained the *iucA*_*29* despite overall module conservation.

The *iroB*_*23*-IS*2* module, another frequently occurring plasmid-borne structure, was distributed across four species: *E. coli* (687/722, 95.2%)*, S. enterica* (17/722, 2.4%)*, K. pneumoniae* (14/722, 1.9%), and *S. flexneri* (4/722, 0.6%). Notably, this module frequently co-occurred with IS*1A*-*iucA*_*45*-IS*1A* (514/722, 71.2%). Recombination analysis of representative plasmids from each species showed nearly identical sequences within this hypervirulence module (Figure 6), suggesting functional maintenance despite phylogenetic distance.

The remaining three high-frequency hypervirulence modules exhibited even more patterns: IS*Kqu3*-*rmpA2*-*iucA_1*-IS*102* and IS*102*-*rmpA-peg-344-iroB_1*-IS*1A* were often present simultaneously at distinct sites on the same plasmid. Their physical linkage formed the composite module IS*Kqu3*-*rmpA2*-*iucA_1*-IS*102*-*rmpA*-*peg-344-iroB_1*-IS*1A*, supporting a modular, stepwise assembly mechanism for virulence acquisition. Comparative analysis of representative strains from all six species carrying this composite module showed >95% sequence similarity across both the module and its flanking regions (Fig 7).

**3.Cross-species transmission of high-frequency HvAMs on chromosomes**

The *iucA_38*-IS*1203E* module was predominantly restricted to *E. coli* (794/796, 99.7%), with only one chromosomally encoded occurrence each identified in *E. hormaechei* and *S. flexneri*. Recombination analysis between the representative *E. coli* strain GCA_002012205.1 and the chromosomes of *E. hormaechei* and *S. flexneri* revealed high genome-wide similarity and coverage (*E. hormaechei*: 97.72% identity, 89.11% coverage; *S. flexneri*: 97.71% identity, 89.13% coverage) (S8 Fig). An extended region of complete sequence identity was observed encompassing the hypervirulence module and its flanking regions.

The IS*Ec40*-*iroB_31*-IS*200C*-IS*Ec13* module, another recurrent structure, was exclusively identified in *E. coli*. A subset of these modules co-occured with *iucA_38*-IS*1203E*. Recombination analysis confirmed that this module can stably integrate into the *E. coli* chromosome, exhibiting high sequence conservation (identity: 98.17%; coverage: 93.14%) (S9 Fig).

Notably, hypervirulence modules including IS*Kqu3*-*rmpA2-iucA_1*-IS102, IS*102*-*rmpA-peg-344-iroB_1*-IS*1A*, and IS*Kqu*3-*rmpA2-iucA_1*-IS*102*-*rmpA-peg-344-iroB_1*-IS*1A* (which may lack certain virulence genes or IS elements) were also detected on chromosomes, albeit at lower frequencies. The IS*Kqu3*-*rmpA2-iucA_1*-IS*102* module occurred exclusively on the *K. pneumoniae* chromosome, while IS*102*-*rmpA-peg-344-iroB_1*-IS*1A* and IS*Kqu3*-*rmpA2-iucA_1*-IS*102*-*rmpA-peg-344-iroB_1*-IS*1A* were sporadically identified on *K. quasipneumoniae* chromosomes. This observation is consistent with the strong species specificity of *rmpA*, *rmpA2*, and *peg-344* for *K. pneumoniae*. Recombination analysis confirmed that these modules can be stably maintained chromosomally across species. The submodules IS*Kqu3*-*rmpA2-iucA_1*-IS*102*, and IS102-*rmpA-peg-344-iroB_1*-IS*1A* were capable of independent dissemination, and their physical linkage formed the composite IS*Kqu3*-*rmpA2-iucA_1*-IS*102*-*rmpA-peg-344-iroB_1*-IS*1A* module. Furthermore, we identified a *K. pneumoniae* strain (GCA_002870865.1) carrying an identical IS*Kqu3*-*rmpA2-iucA_1*-IS*102*-*rmpA-peg-344-iroB_1*-IS*1A* module on both its chromosome and a resident plasmid (Supplementary Figure 6), demonstrating plasmid-to-chromosome transfer of this hypervirulence module, which may facilitate stable vertical inheritance within bacterial populations.

A distinct hypervirulence module, IS*100kyp*-*iroB_6-peg-344-rmpA*-IS*Kpn1/IS102*, was identified primarily on the *K. pneumoniae* chromosome, with a single exception detected in *K. variicola*. Recombination analysis of two *K. pneumoniae* chromosomes (GCA_002163895.1, GCA_009497695.1) and one *K. variicola* chromosome (GCA_900636185.1) revealed a conserved genomic segment containing this module, supporting its role as a stable mobile unit. This module typically occurred independently, suggesting it constitutes a unique hypervirulence module with a distinct evolutionary trajectory separate from the IS*Kqu3*-*rmpA2-iucA_1*-IS*102*-*rmpA-peg-344-iroB_1*-IS*1A* module (S7 Fig).
